# Evaluation of uncharacterized quinoa (*Chenopodium quinoa* Willd.) accessions for salinity tolerance during seedling emergence and early growth

**DOI:** 10.3389/fpls.2026.1856220

**Published:** 2026-06-17

**Authors:** Jhaman Das Suthar, Girisha K. Ganjegunte, Rahmatullah Hashimi

**Affiliations:** Soil and Crop Sciences, Texas A&M AgriLife Research, Texas A&M University System, El Paso, TX, United States

**Keywords:** facultative halophyte, saline irrigation water, salt-tolerance, seedling emergence, superfood quinoa

## Abstract

**Introduction:**

Salinity stress is a major constraint to crop establishment and productivity in arid and semi-arid regions, necessitating the identification of salt-tolerant crops and accessions.

**Methods:**

This greenhouse study assessed the salinity tolerance of 22 quinoa (*Chenopodium quinoa* Willd.) accessions, including uncharacterized accessions, seedling emergence, and early seedling growth under six irrigation water salinity levels (ECiw ≈ 1, 3, 6, 9, 12, and 15 dS m^-1^). A completely randomized design with three replications was employed. Salinity tolerance was assessed using the salinity tolerance index (STI) and membership function value (MFV), and accessions were classified through hierarchical cluster analysis.

**Results:**

Increasing salinity significantly reduced seedling emergence, delayed emergence, and decreased growth and biomass (P ≤ 0.01). At 15 dS m^-1^, emergence declined by 66.7%, and biomass decreased by more than 90%. Salinity altered ion accumulation, increasing Na^+^ and Ca^2+^ concentrations while decreasing K^+^ concentration and the ionic ratios (K^+^/Na^+^ and Ca^2+^/Na^+^). Among the accessions, PI 634923 exhibited the highest salinity tolerance, followed by PI 698783, PI 698773, PI 698780, and PI 698775. Biomass-related traits showed stronger associations with overall tolerance than seedling emergence traits. The threshold salinity corresponding to 50% STI was estimated at approximately 10.5 dS m^-1^ for seedling emergence and the early growth stage.

**Discussion:**

Overall, the study reveals substantial genetic variability in quinoa and identifies promising accessions for saline environments. The inclusion of previously uncharacterized germplasm provides novel insights into breeding programs and supports the development of climate-resilient cropping systems.

## Highlights

- PI 634923 showed the highest tolerance across all salinity levels.
- Biomass traits were more sensitive indicators than seedling emergence traits.
- MFV with cluster analysis effectively assessed salinity tolerance.
- Threshold salinity for 50% STI at the seedling stage was ≈ 10.5 dS m^-1^.

## 1 Introduction

Global agriculture faces mounting challenges from climate change, freshwater scarcity, and soil salinization, especially in arid and semi-arid regions that cover nearly one-third of the Earth’s land surface (ICARDA, 2021). Rising temperatures have intensified evaporative demand, while increasingly variable rainfall patterns are reducing the availability and reliability of irrigation water. Growing urban and industrial demands further limit agricultural access to freshwater resources (Chaganti et al., 2021). These pressures are expected to intensify in the coming decades, posing serious risks to agricultural productivity and food security, particularly in regions dependent on limited irrigation supplies (Sun et al., 2013; Jain et al., 2024). Under these conditions, irrigation often relies on marginal-quality water with elevated salt concentrations, leading to progressive salt accumulation in the soil profile (Chhabra, 2022). This accumulation adversely affects plant growth and productivity, becoming a major constraint on sustainable agriculture. The problem is particularly severe in arid systems, where limited natural leaching, poor soil fertility, and high evapotranspiration accelerate salt buildup in the root zone (Naorem et al., 2023; Derbew et al., 2026). Therefore, identifying crops capable of maintaining productivity under saline and water-limited conditions is essential for sustainable agriculture.

Salinity stress affects crops throughout their life cycle; however, its impact is most critical during seedling emergence and early seedling growth, when successful establishment determines final crop productivity (Läuchli and Grattan, 2007). At this stage, plants are highly sensitive to osmotic stress and ion toxicity, which reduce water uptake, delay metabolic processes, and impair seedling vigor. Quinoa (*Chenopodium quinoa* Willd.) has emerged as a promising crop for such environments due to its facultative halophytic nature and tolerance to salinity, drought, and heat stress (Adolf et al., 2013; Chaganti and Ganjegunte, 2024; Vidal et al., 2026). In addition to its stress tolerance, quinoa is widely recognized as a nutrient-rich crop with a balanced amino acid profile and high concentrations of essential micronutrients (Mohamed et al., 2021; Olivera et al., 2022). These attributes have enhanced their global importance as a climate-resilient and nutritionally valuable crop.

In the United States, quinoa cultivation has expanded in the southwestern regions of New Mexico and Colorado, where it is being explored as a high-value alternative to traditional crops such as cotton and alfalfa. Similarly, in regions including the Middle East, North Africa, and Central Asia, quinoa has demonstrated strong potential under saline irrigation and water-limited conditions (Bazile et al., 2016; Eisa et al., 2017). Despite its adaptability, quinoa exhibits considerable genetic variability in response to salinity stress, particularly during early growth stages that are critical for establishment and yield formation (Adolf et al., 2013; Böhm et al., 2018).

High salinity reduces water availability to seeds, delaying emergence, while excessive ion accumulation can damage seed tissues and embryos (Llanes et al., 2016). Maintenance of ionic balance, particularly higher K^+^/Na^+^ and Ca²^+^/Na^+^ ratios, is a key determinant of salinity tolerance, as these ratios regulate osmotic adjustment, enzyme activity, and membrane stability (Ashraf and Munns, 2022; Turan et al., 2022). Potassium plays a vital role in osmotic regulation and metabolic functions, whereas calcium contributes to membrane stabilization and the regulation of ion transport (Putra et al., 2026). In arid and semi-arid environments, low rainfall combined with high evaporation often leads to salt accumulation at the soil surface, where seeds are sown, intensifying the adverse effects on germination and early seedling growth (Castañeda-Loaiza et al., 2024). Consequently, the early growth stage provides a critical window for evaluating salinity tolerance and identifying genotypes capable of successful establishment under saline conditions.

Evaluating genotypic variation under controlled conditions is essential for accurately assessing plant responses to salinity stress. Greenhouse experiments enable precise control of salinity levels and minimize environmental variability, allowing reliable comparisons of seedling emergence and its growth traits. However, information on early-stage salinity tolerance in quinoa, particularly during seedling emergence, remains limited. Seedling emergence is a critical yet often underexplored phase for successful crop establishment. Moreover, systematic evaluation of diverse, regionally relevant quinoa germplasm, especially previously uncharacterized accessions, remains scarce under controlled salinity conditions. Most previous studies have focused on a limited number of well-known genotypes or later growth stages, leaving a significant knowledge gap regarding the variability and adaptive potential of unexplored genetic resources during early growth. Notably, the present study evaluates 22 quinoa accessions, including previously uncharacterized accessions not assessed in earlier salinity tolerance studies, thereby providing novel insights into unexplored genetic diversity and improving understanding of quinoa adaptation mechanisms under saline environments.

In this context, the present study focuses on a diverse set of quinoa accessions from the southwestern United States and South America, evaluated under controlled salinity conditions. The study emphasizes early-stage growth responses and employs a systematic approach to assess variability in salinity tolerance across multiple traits and indices. Therefore, the objectives of this study were to (i) evaluate differences among quinoa accessions in seedling emergence potential and early seedling growth under varying salinity levels, (ii) identify salt-tolerant accessions, and (iii) classify accessions into distinct tolerance groups using integrated indices. Overall, this research addresses critical knowledge gaps in quinoa cultivation under saline conditions by focusing on early-stage performance and genetic variability among diverse accessions. The identification of salinity tolerance in previously uncharacterized genotypes provides valuable genetic resources for breeding programs and supports the development of climate-resilient cropping systems in salt-affected environments.

## 2 Materials and methods

### 2.1 Experimental design and salinity treatments

A greenhouse study was conducted at the Texas A&M AgriLife Research Center in El Paso, Texas (31°41′47.5″ N, 106°16′57.6″ W) to assess the salinity tolerance of quinoa accessions during the early growth stage. Seeds from 22 quinoa accessions, obtained from the USDA National Plant Germplasm System (https://npgsweb.ars-grin.gov/gringlobal/search), were used in this study. Detailed information about the accessions is provided in **Table 1**. The experiment followed a completely randomized design with two factors: quinoa accession and irrigation water salinity. Six salinity levels were tested, including freshwater as the control (ECiw ≈ 1 dS m^-1^) and saline irrigation water with electrical conductivities of 3, 6, 9, 12, and 15 dS m^-1^. Saline solutions were prepared in the laboratory by dissolving specific amounts of NaCl in freshwater to achieve the target electrical conductivity levels. The freshwater used for the control treatment was collected from the Rio Grande River, which generally contains a mixture of salts with varying ionic compositions. The chemical characteristics of the irrigation water are presented in **Table 2**. Each treatment combination was replicated three times.

**Table 1:** Description and origin of quinoa accessions used in this study.

A#	USDA PI no.	Plant name/accession	Origin
1	PI 698768	KASLAEA	New Mexico, United States
2	PI 698769	PISON	New Mexico, United States
3	PI 698771	COPACABANA	New Mexico, United States
4	PI 698772	32ALC	New Mexico, United States
5	PI 698773	A5P	New Mexico, United States
6	PI 698774	21 GR	New Mexico, United States
7	PI 698775	23TES	New Mexico, United States
8	PI 698776	16 GR	New Mexico, United States
9	PI 698777	107R	New Mexico, United States
10	PI 698779	101R	New Mexico, United States
11	PI 698780	105R	New Mexico, United States
12	PI 698781	29TES	New Mexico, United States
13	PI 698782	20ALC	New Mexico, United States
14	PI 698783	22 GR	New Mexico, United States
15	PI 698784	177R	New Mexico, United States
16	PI 596293	COLORADO 407D	Colorado, United States
17	PI 614880	CHADMO	Los Lagos, Chile
18	PI 614925	CQ 125	La Paz, Bolivia
19	PI 634919	PICHAMAN	Chile
20	PI 634923	UDEC-1	Chile
21	PI 677096	537 BK60-B	Maryland, United States
22	PI 677097	PLANT VIRUS	South Carolina, United States

**Table 2:** Chemical characteristics of freshwater (control=S0) and saline irrigation water used in the study.

Variable	Salinity levels (dS m^-1^) of irrigation water					
	1.00	3.00	6.00	9.00	12.00	15.00
Measured ECiw (dS m^-1^)	0.74	3.08	6.12	9.33	12.32	15.45
pH	7.70	7.21	7.13	7.05	7.04	7.04
SAR, (mmol L^-1^)^1/2^	1.46	10.12	27.65	48.94	49.82	73.03
Soluble ions (mg L^-1^)						
Na^+^	2.87	20.32	52.87	93.76	114.14	144.20
Mg^2+^	0.59	0.59	0.23	0.00	0.59	0.35
Ca^2+^	3.26	3.44	3.43	3.67	4.66	3.55
Cl^-^	3.91	25.95	51.87	85.49	110.88	137.91
SO_4_^2-^	0.22	0.24	0.23	0.24	0.22	0.23

### 2.2 Potting substrate preparation and seed sowing

Traditional potting mix and peat moss were sieved to remove large plant particles, then mixed at a 2:1 (v/v) ratio. The prepared substrate was used to fill plug trays containing 200 cells, each with a volume of 15 cm³. Before filling the trays, the substrate was pre-moistened with the respective saline water solution according to the treatment levels. Following the experimental design described above, different accessions were arranged within each tray. For each treatment combination (accession × salinity), 20 seeds were sown (one per cell) at approximately 1 cm depth. Each replication consisted of an independent set of 20 cells per treatment combination, serving as the experimental unit. Multiple accessions (up to 10) were accommodated within a single tray by assigning separate groups of 20 cells to each accession in each replication. Due to the short duration of the experiment (15 days after sowing), no visible root growth restriction was observed in the plug cells. After sowing, trays were monitored daily, and moisture was maintained by spraying the respective saline water solutions throughout the experimental period.

### 2.3 Seedling emergence and biomass determination

Seedling emergence was recorded daily for 15 days. Seedling emergence percentage (SE%) was calculated using Equation 1 (Suthar et al., 2025). Mean Seedling Emergence Time (MSET) and the coefficient of variation of seedling emergence time (CVt) were determined according to Ranal et al. (2009) using Equations 2 and 3, respectively.

(1) $$\text{SE}\%=\frac{\text{Number of seedling emerged}}{\text{Total seed sown}}\times 100$$

(2) $$\text{MSET}=\frac{\sum_{i=1}^{k}n_{i}t_{i}}{\sum_{i=1}^{k}n_{i}}$$

(3) $$\text{CVt}=\frac{\text{S}t}{MSET}\times 100$$

where MSET is the mean seedling emergence time (days), *n_i_* is the number of seeds that emerged on the *i*-th day, *t_i_* is the time in days from sowing to the *i*-th observation, *k* is the observation on the last day. CVt represents the Coefficient of variation of seedling emergence time, and *St* is the standard deviation of MSET.

Fifteen days after sowing, seedlings were harvested, and their shoot length and fresh weight were measured. The seedlings were then dried in an oven at 65 °C until a constant weight was obtained, after which the dry biomass was determined.

### 2.4 Potting substrate, irrigation water, and shoot tissue analysis

Potting substrate samples were collected after mixing with saline water and were air-dried before analysis. Substrate samples were extracted with deionized (D.I.) water at a 1:10 substrate-to-water ratio following the procedures developed by the U.S. Composting Council (2002), as shown in **Table 3**. Artificially prepared saline water and freshwater (control) were also sampled. Irrigation water and substrate extracts were analyzed for electrical conductivity (EC; Rhoades, 1996) and pH (Thomas, 1996) using a Fisher Scientific Accumet XL600 dual-channel benchtop digital meter. Concentrations of soluble cations (Ca^2+^, Mg^2+^, K^+^, and Na^+^) and anions (Cl^-^ and SO_4_^2-^) were determined using ion chromatography following the methods of Suarez (1996) and Tabatabai and Frankenberger (1996). Sodium adsorption ratio (SAR) was calculated following Essington (2003). Shoot concentrations of Na^+^, K^+^, and Ca^2+^ were determined using inductively coupled plasma-optical emission spectrometry (ICP-OES) following the U.S. EPA method (1996).

**Table 3:** Chemical properties of the potting substrate (1:10) extract after irrigation water treatments.

Variable	Salinity levels (dS m^-1^) of irrigation water					
	1.00	3.00	6.00	9.00	12.00	15.00
EC (dS m^-1^)	2.47	3.50	6.41	9.28	12.68	15.66
pH	5.54	5.88	5.50	5.41	5.52	5.18
SAR, (mmol L^-1^)^1/2^	2.95	4.79	12.90	11.28	17.28	22.22
Soluble ions (mg L^-1^)						
Na^+^	225.94	446.66	1248.30	1434.62	2271.25	3194.90
K^+^	105.95	118.66	33.05	104.28	66.24	58.92
Mg^2+^	228.89	358.30	374.51	646.68	706.19	830.46
Ca^2+^	60.24	67.76	84.54	157.23	148.38	176.33
Cl^-^	339.45	643.04	2166.27	2435.22	3965.95	4684.83
NO_3_^-^	131.83	341.27	276.31	487.36	465.12	493.37
PO_4_^3-^	46.45	41.09	39.09	73.56	70.25	75.07
SO_4_^2-^	209.87	237.10	236.49	392.41	454.31	432.37

### 2.5 Salt tolerance evaluation

Initially, the salinity tolerance index (STI) was calculated according to Anwar et al. (2026) using Equation 4:

(4) $$STI=\frac{X_{c}\times X_{s}}{{\left({\bar{X}}_{c}\right)}^{2}}$$

where *X_c_* and *X_s_* represent the trait values of each accession at a given salinity level under control and saline conditions, respectively, and ${\bar{X}}_{c}$ is the mean trait value under control conditions across all accessions.

The STI was calculated separately for each accession at each salinity level prior to further analysis. Salt tolerance of quinoa accessions was further evaluated using the membership function value (MFV) based on the fuzzy comprehensive evaluation method (Chen et al., 2012). The MFV for salt tolerance was calculated using the following Equation 5:

(5) $$X_{i}=\frac{X-X_{\text{min}}}{X_{\text{max}}-X_{\text{min}}}$$

where *X_i_* is the MFV of the STI for a specific accession, *X* is the observed STI value of that accession, and *X*_max_ and *X*_min_ are the maximum and minimum STI values observed among all accessions, respectively (Ding et al., 2018).

Salt tolerance of the accessions was evaluated based on the average MFV of each trait. The MFV values ranged from 0 to 1, where higher values indicate greater salt tolerance. For each accession, the mean MFV was calculated as the average of the MFVs for seedling emergence (SE) %, shoot length (ShL), seedling fresh weight (SFW), and seedling dry weight (SDW) across salinity levels (3, 6, 9, 12, and 15 dS m^-1^). Therefore, each accession had a unique average MFV, and accessions with higher mean MFV values were considered more salt-tolerant.

### 2.6 Statistical analysis

All data were analyzed using IBM SPSS Statistics for Windows (Version 29.1.1; IBM Corp., Armonk, NY, USA). Treatment effects were initially evaluated using analysis of variance (ANOVA). Subsequently, a Generalized Linear Mixed Model (GLMM) was employed to assess the main effects of salinity and quinoa accession, as well as their interaction. Both salinity and quinoa accession were treated as fixed factors, and their interaction term was included to capture treatment-specific responses. When significant differences were detected, mean separation was performed using Tukey’s Honest Significant Difference (HSD) *post hoc* test at *p* ≤ 0.05 under the assumption of equal variances. Statistical significance was set at p< 0.05 for all traits. Additionally, hierarchical cluster analysis was conducted to assess salinity tolerance among quinoa accessions. Using the dendrogram produced by Ward’s linkage method and squared Euclidean distance, accessions were categorized into five distinct salinity tolerance groups: highly salt-tolerant (HST), salt-tolerant (ST), moderately salt-tolerant (MST), salt-sensitive (SS), and highly salt-sensitive (HSS). This classification was based on four key traits: seedling emergence (SE%), shoot length (ShL), seedling fresh weight (SFW), and seedling dry weight (SDW), with accessions labeled accordingly. The clustering pattern, interpreted from the rescaled distance, clearly separated accessions into distinct groups based on their different responses to salinity stress. Furthermore, correlation analysis and linear regression were performed in Microsoft Excel (Microsoft Corporation, Redmond, WA, USA) to evaluate relationships among measured traits and estimate salinity threshold values.

## 3 Results

### 3.1 Seedling emergence response to salinity

Seedling emergence (SE%), mean seedling emergence time (MSET), and variability in emergence uniformity (CVt) across salinity levels and accessions are presented in **Supplementary Table 1A–C**. Both salinity and accession, and their interaction, significantly affected all three parameters (P ≤ 0.01). As salinity increased, seedling emergence declined markedly, with average SE% dropping from 60.8% under control conditions to 20.2% at 15 dS m^-1^, representing a 66.7% reduction. Furthermore, **Table 4** shows that maximum SE remained relatively high (83.3%) even at 15 dS m^-1^, indicating considerable variability in quinoa salinity tolerance. Correspondingly, the mean seedling emergence time increased from 6.5 to 7.4 days, indicating a delay in emergence. In contrast, CVt slightly declined from 17.0 to 13.1, with substantial accession × salinity variation persisting. Among the accessions, PI 634923 consistently performed best across salinity levels, maintaining high emergence (96.7–83.3%) and the lowest MSET (4.6–5.5 days), indicating rapid and stable emergence. Notably, PI 614880 (70.0–41.7% SE) and PI 698783 (91.7–35.0% SE) also performed relatively well under salinity stress. By comparison, accessions such as PI 698782 and PI 677096 had complete inhibition at ≥12 dS m^-1^, reflecting high sensitivity. Trends in emergence uniformity further supported these results: PI 614880 (13.0–15.9) and PI 698783 (12.7–17.1) had lower CVt values. Meanwhile, more sensitive accessions, such as PI 614925, showed greater variability, with CVt up to 22.4. Overall, across accessions, PI 634923 demonstrated superior tolerance, with higher emergence, faster emergence, and better uniformity under saline conditions.

**Table 4:** Mean, minimum, and maximum values of seed emergence percentage (SE), shoot length (ShL), shoot fresh weight (SFW), and shoot dry weight (SDW) among 22 quinoa accessions grown under different salinity levels.

Salinity levels (dS m^-1^)	SE (%)			ShL (cm)			SFW (mg/seedling)			SDW (mg/seedling)		
	Mean	Min.	Max.	Mean	Min.	Max.	Mean	Min.	Max.	Mean	Min.	Max.
1	60.8a	13.3	96.7	5.0a	2.6	7.1	51.87a	23.3	79.5	6.2a	3.0	11.4
3	58.0a	15.0	95.0	4.3ab	2.8	5.5	40.46b	21.6	59.3	4.5b	2.0	7.8
6	50.3b	10.0	93.3	4.5b	1.6	6.2	35.42b	16.3	53.3	3.6b	0.5	6.2
9	45.2ab	0.0	96.7	4.1b	2.2	5.5	24.44c	10.4	37.5	1.7c	0.5	4.1
12	28.3bc	0.0	88.3	2.5c	1.7	3.2	20.49c	3.0	36.0	1.7c	0.6	6.4
15	20.2c	0.0	83.3	2.1d	1.2	3.0	11.91d	10.0	15.0	0.7c	0.6	1.0

Means followed by different letters differ significantly at the 0.05 level.

### 3.2 Seedling growth and biomass response to salinity

Shoot length and seedling fresh and dry weight across salinity levels and accessions are presented in **Supplementary Table 1D–F**. Growth parameters were significantly affected by salinity (S), accession (A), and their interaction (S × A) (P ≤ 0.01). Seedling growth and biomass declined markedly as salinity increased. This decline was shown in **Table 4**. Mean shoot fresh weight dropped from 51.87 to 11.91 mg seedling^-1,^ and shoot dry weight fell from 6.2 to 0.7 mg seedling^-1^ as salinity rose from 1 to 15 dS m^-1^. At the control, the average shoot length was 5.0 cm. At 15 dS m^-1^, shoot length dropped to 0.5 cm, a 90.4% reduction. Among accessions, PI 634923 maintained the greatest shoot length at 15 dS m^-1^ (2.5 cm), and PI 698780 reached 2.0 cm. All other accessions showed nearly complete inhibition of shoot growth at this salinity. Fresh weight at 15 dS m^-1^ was highest in PI 634923 (15.0 mg seedling^-1^), followed by PI 698780 (12.0 mg) and PI 614880 (11.6 mg). Most other accessions showed negligible or zero biomass. For dry weight, PI 698780 led (1.0 mg seedling^-1^), followed by PI 614880 (0.7 mg) and PI 634923 (0.6 mg). Overall, PI 634923, PI 614880, and PI 698780 demonstrated the greatest tolerance to high salinity and outperformed the other accessions.

### 3.3 Salinity-induced changes in Na^+^, K^+^, Ca^2+^ accumulation, and ionic ratios in quinoa accessions

Salinity stress significantly affected the ion accumulation and ionic balance in quinoa accessions (p<0.01; **Supplementary Table 1**). Specifically, Na^+^ and Ca^2+^ concentrations increased progressively with salinity, whereas K^+^ concentration and ionic ratios (K^+^/Na^+^ and Ca^2+^/Na^+^) decreased. When comparing salinity levels, the lowest average values of Na^+^ and Ca^2+^ (0.92 and 0.33 mmol g^-1^) were observed under control conditions (1 dS m^-1^). In contrast, the highest average values (5.34 and 1.1 mmol g^-1^) were observed at elevated salinity levels (12–15 dS m^-1^), respectively. K^+^ concentration was also affected, ranging from 2.0–2.8 mmol g^-1^ at low salinity (1 and 3 dS m^-1^) to 0.44–2.0 mmol g^-1^ at higher salinity levels (9–15 dS m^-1^). Notably, some accessions (PI 634923 and PI 698780) maintained relatively high K^+^ and Ca^2+^ levels even under severe stress, suggesting greater ion selectivity and retention. Furthermore, the ionic ratios (K^+^/Na^+^ and Ca^2+^/Na^+^) decreased sharply with increasing salinity, with higher ratios (up to 4.75 and 0.69) observed at 1–3 dS m^-1^ and much lower values (0.16 and 0.05) at 12–15 dS m^-1^. Overall, these results highlight strong genotypic variation in ion regulation and salinity tolerance among quinoa accessions.

### 3.4 Trait-based relationships of salinity tolerance and determination of threshold salinity (ECiw)

**Figure 1:**
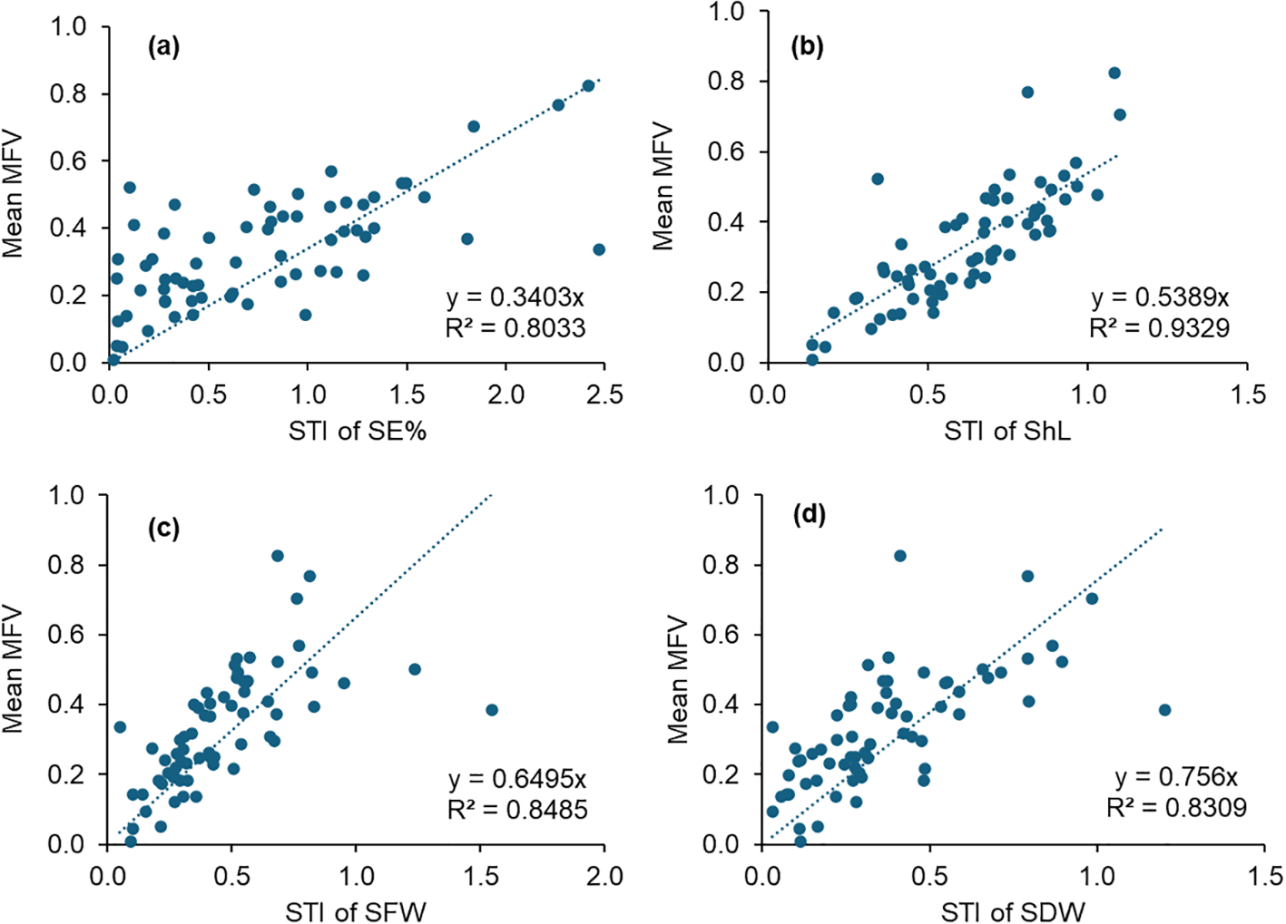
Linear relationships between mean MFV (across traits) and STI of **(A)** SE%, **(B)** ShL, **(C)** SFW, and **(D)** SDW for each accession, with fitted regression lines.

**Figure 2:**
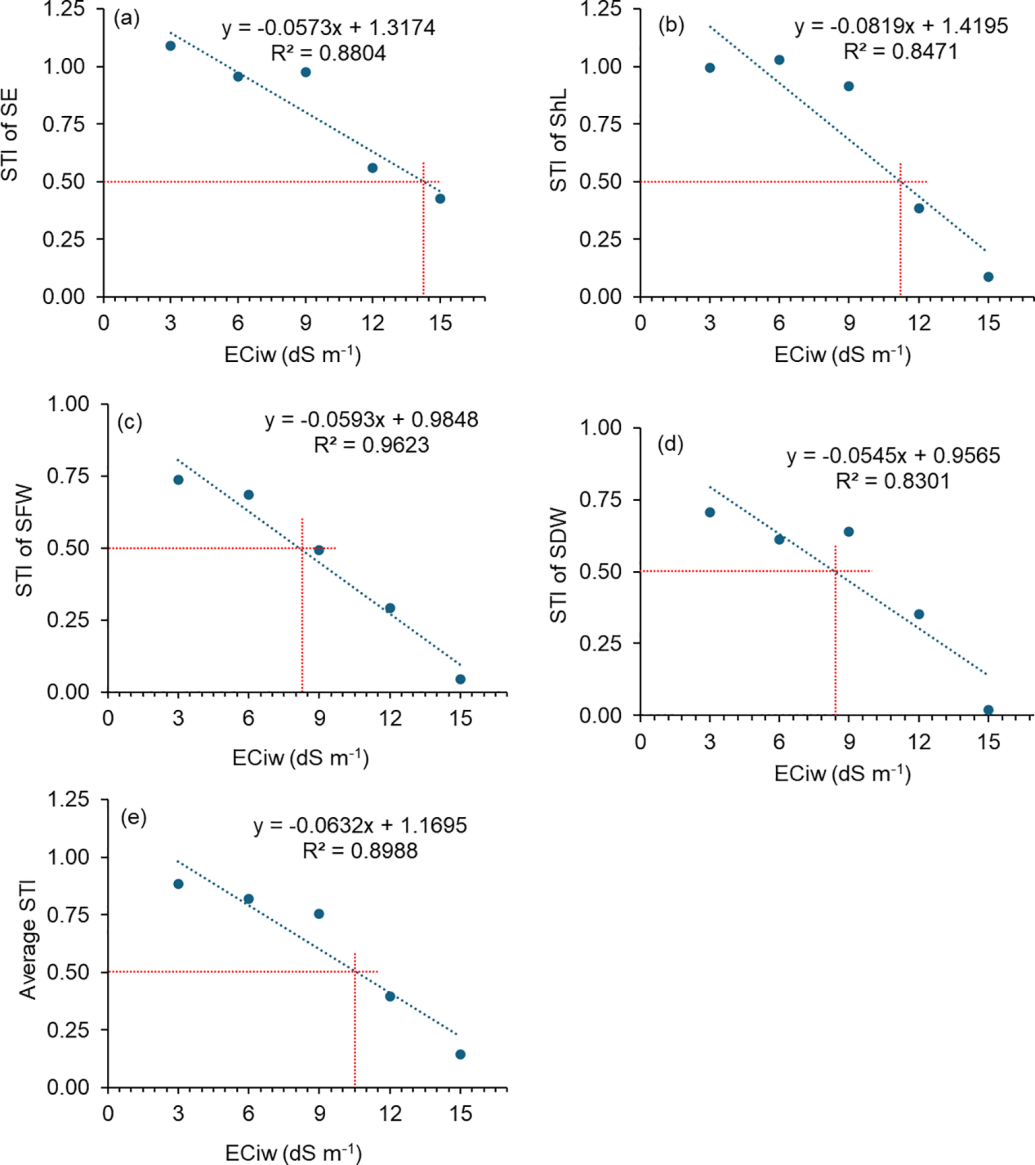
Determination of threshold salinity of irrigation water (ECiw) based on 50% salinity tolerance index (STI) for SE **(A)**, ShL **(B)**, SFW **(C)**, SDW **(D)**, and mean STI **(E)** in quinoa accessions (n = 22).

The relationships between the salinity tolerance index (STI) of individual traits and membership function value (MFV) across traits are presented in **Figure 1**. A positive linear relationship was observed between STI and mean MFV for all evaluated traits, indicating that accessions with higher STI values consistently exhibited superior performance under salinity stress. Seedling fresh weight (SFW) and seedling dry weight (SDW) showed stronger associations with mean MFV than seedling emergence percentage (SE%) and shoot length (ShL), suggesting biomass-related traits contributed more to overall salinity tolerance. These results demonstrate that STI is a reliable indicator for identifying salt-tolerant accessions based on integrated trait performance. The threshold salinity of irrigation water (ECiw) corresponding to 50% STI for individual traits and mean STI is illustrated in **Figure 2**. A clear negative linear relationship was observed between STI and ECiw, indicating a progressive decline in plant performance as salinity increases. The ECiw thresholds varied among traits, reflecting differences in sensitivity to salinity. Seedling emergence percentage (SE%) maintained 50% STI at higher ECiw levels, indicating greater tolerance during this stage, whereas shoot length (ShL), seedling fresh weight (SFW), and seedling dry weight (SDW) declined more steeply, suggesting greater sensitivity of growth and biomass traits. The mean STI provided an integrated assessment across all traits and showed a consistent ECiw threshold. Based on mean STI, the 50% tolerance level was reached at approximately 10.5 dS m^-1^, representing the overall salinity tolerance threshold for quinoa accessions under the studied conditions. These results indicate that increasing salinity significantly reduces plant performance. The derived ECiw threshold based on 50% STI provides a practical benchmark for defining salinity limits for quinoa cultivation under saline irrigation.

### 3.5 Quinoa accession variation in salt tolerance

**Figure 3:**
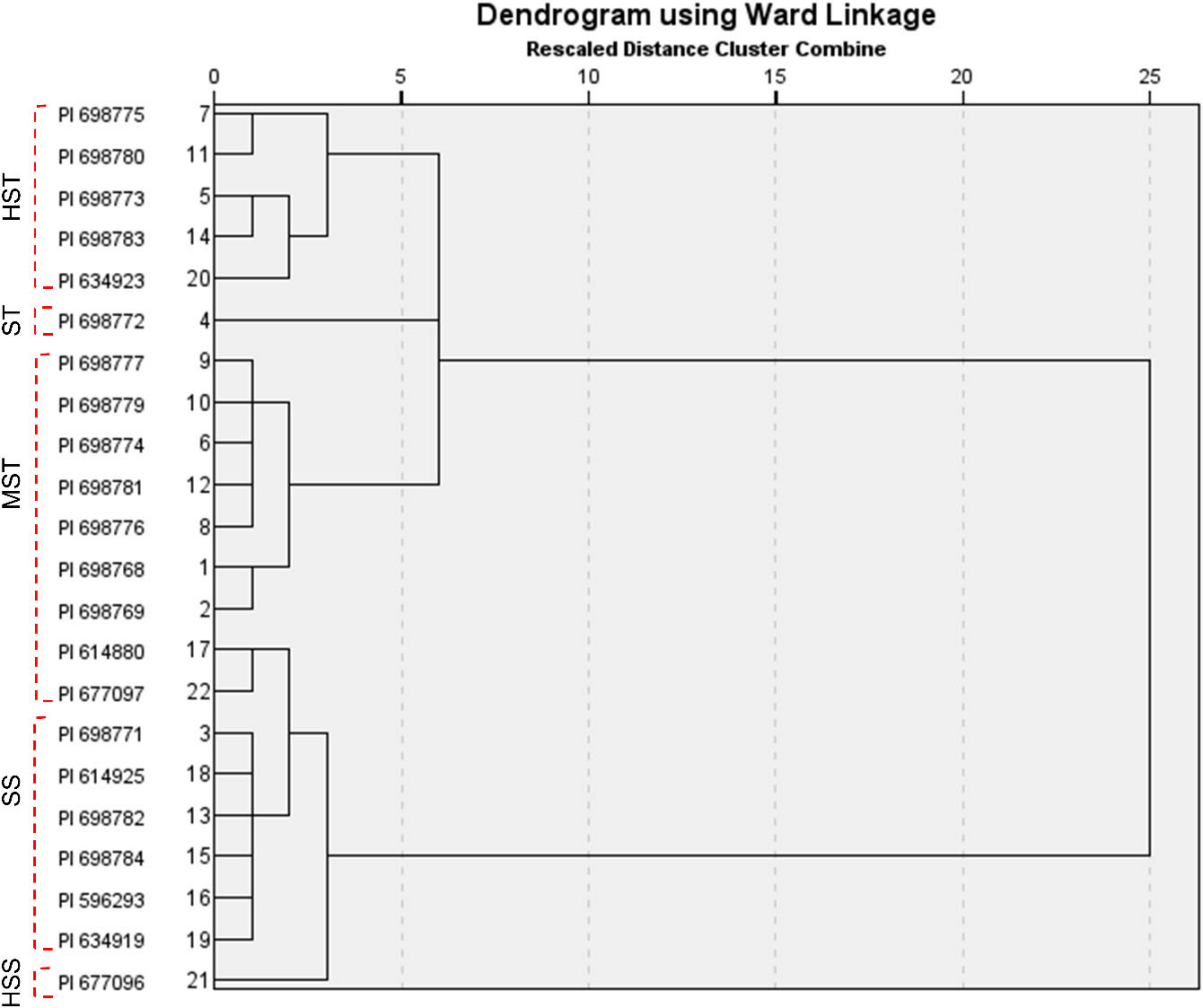
Hierarchical cluster dendrogram of quinoa accessions based on seedling emergence and growth traits under salt stress conditions. HST, ST, MST, SS, and HSS represent highly salt-tolerant, salt-tolerant, moderately salt-tolerant, salt-sensitive, and highly salt-sensitive accessions, respectively.

Hierarchical cluster analysis clearly differentiated the 22 quinoa accessions into five distinct groups, representing varying levels of salinity tolerance (**Figure 3**). The clustering pattern revealed significant genotypic variation in response to salt stress, as indicated by seedling emergence, growth, and biomass traits. The highly salt-tolerant (HST) group comprised accessions PI 698775, PI 698780, PI 698773, PI 698783, and PI 634923, which clustered closely together because of their superior performance under increasing salinity, maintaining higher emergence and biomass. The salt-tolerant (ST) group included only PI 698772, indicating comparatively stable performance under salinity stress. The moderately salt-tolerant (MST) group consisted of PI 698777, PI 698779, PI 698774, PI 698781, PI 698776, PI 698768, PI 698769, PI 614880, and PI 677097, showing moderate reductions in growth and seedling emergence traits as salinity increased. In contrast, the salt-sensitive (SS) group included PI 698771, PI 614925, PI 698782, PI 698784, PI 596293, and PI 634919, which exhibited substantial reductions in emergence and biomass under elevated salinity. The highly salt-sensitive (HSS) group was represented solely by PI 677096, which was separated at the greatest rescaled distance, indicating the poorest performance and highest susceptibility to salt stress. Overall, the dendrogram demonstrates clear genetic variability among quinoa accessions and provides a robust classification of salinity tolerance levels for selecting suitable genotypes under saline conditions.

## 4 Discussion

### 4.1 Impact of salinity on early establishment of quinoa

This study clearly demonstrates that salinity stress significantly impairs early growth in quinoa, as evidenced by lower seedling emergence, delayed emergence, and reduced growth and biomass. The observed increase in mean seedling emergence time (MSET) further indicates that salinity delays emergence. This delay is primarily attributable to ion toxicity resulting from excessive salt accumulation during germination and early seedling growth, which can impair seedling establishment and delay emergence (Nikolić et al., 2023). High salt concentrations may also result in uneven germination and greater variability in seedling emergence among accessions. The relatively lower average SE% under control conditions was mainly influenced by a few accessions with inherently low germination potential. In contrast, PI 634923 maintained consistently high emergence under both control and saline conditions. This accession was also classified within the highly tolerant group in the hierarchical cluster analysis. Additionally, variability in seedling emergence time (CVt) reflects underlying genetic variability and differences in salinity tolerance, indicating that some quinoa accessions possess a greater ability to withstand salt stress than others. These genetic differences are considered important factors in quinoa adaptation to saline and harsh environmental conditions (Zhou et al., 2023). Seedling growth parameters proved to be more sensitive to salinity than seedling emergence traits. Shoot length, fresh weight, and dry weight experienced significant reductions, indicating that post-emergence growth is more vulnerable to salt stress. This aligns with recent findings that salinity inhibits cell division and elongation, reduces photosynthetic efficiency, and limits biomass accumulation due to ionic imbalance and nutrient disruption (Ghosh et al., 2025; Huanhe et al., 2024; Santanoo et al., 2023). The greater decline in biomass-related traits than in seedling emergence suggests that early seedling development requires greater metabolic stability, making it more susceptible to salinity stress. Among the evaluated accessions, PI 634923 consistently exhibited higher emergence, faster emergence, and greater biomass under saline conditions. These results indicate stronger adaptive mechanisms, including osmotic adjustment, ion regulation, and efficient stress tolerance pathways. Similar genotypic differences in salinity tolerance have been reported in quinoa and other crops, where tolerant genotypes maintain better growth (Adolf et al., 2013; Hussin et al., 2023). In contrast, sensitive accessions experienced complete inhibition at higher salinity levels, reflecting their inability to manage ionic toxicity and osmotic stress.

### 4.2 Salinity effects on ion accumulation

Salinity markedly altered ion accumulation and ionic balance, reinforcing ionic toxicity as a major driver of quinoa seedling decline. Increasing ECiw elevated Na^+^ and Ca^2+^, while reducing K+, causing steep declines in K^+^/Na^+^ and Ca^2+^/Na^+^ ratios at 12–15 dS m^-1^, likely impairing enzyme activity, osmotic adjustment, and membrane stability (Causin et al., 2020; Wang et al., 2022). The observed increase in Ca^2+^ under salinity conditions may also have been partially influenced by the mineral composition of the growth substrate, which could have affected Ca^2+^ uptake patterns under salt stress. Genotypic variation in ion management underpinned differences in tolerance: tolerant accessions (PI 634923; PI 698780) maintained higher K^+^ and Ca^2+^ levels under stress. As a result, the observed K^+^ retention, Na^+^ exclusion, and Ca^2+^ associated stability in tolerant accessions are consistent with more selective ion transport and membrane-stabilizing mechanisms. In contrast, higher Na^+^ and lower K^+^/Na^+^ and Ca^2+^/Na^+^ in sensitive accessions likely reflect weaker ion selectivity and disrupted ionic homeostasis, leading to growth failure (Dudev and Lim, 2010; Kiani et al., 2015).

### 4.3 Salinity tolerance and classification of quinoa accessions

The integration of the salinity tolerance index (STI) and the membership function value (MFV) proved effective for evaluating and classifying quinoa accessions based on their overall performance under salinity stress. The positive relationship between STI and MFV (**Figure 1**) indicates that both indices reliably reflect accessions’ tolerance potential, with higher STI values corresponding to a greater ability to sustain growth under saline conditions. Similar approaches have been widely used in recent studies, where MFV integrates multiple physiological and growth traits to provide a comprehensive assessment of salt tolerance across germplasms (Gyanagoudar et al., 2024; Kaur et al., 2022; Wu et al., 2019). Biomass-related traits (seedling fresh and dry weight) exhibited stronger associations with MFV than seedling emergence traits, suggesting that these parameters are more reliable indicators of salinity tolerance. This is consistent with recent findings showing that biomass accumulation is closely linked to physiological performance under salt stress, including osmotic adjustment and ion regulation (Anwar et al., 2025; Huanhe et al., 2024; Zuo et al., 2025). Therefore, traits related to fresh and dry weight are often considered key selection criteria for identifying salt-tolerant genotypes at early growth stages (Javed et al., 2022; Rai et al., 2026).

The determination of threshold salinity (ECiw ≈ 10.5 dS m^-^¹ at 50% STI) provides a practical benchmark for the early growth stage of quinoa. The regression equations presented in **Figure 2** further demonstrated strong relationships between ECiw and STI across all evaluated traits. Notably, seedling emergence traits maintained 50% STI at relatively higher salinity levels (up to approximately 14.5 dS m^-1^), whereas biomass-related traits declined below the 50% threshold at ECiw values lower than 9 dS m^-1^. This finding clearly indicates that seedling emergence processes are comparatively more tolerant to salinity stress than post-emergence biomass accumulation. The use of a 50% reduction criterion is widely accepted in recent studies as an optimal threshold to differentiate tolerant and sensitive genotypes, as it ensures sufficient stress expression while maintaining plant viability (Ahmed et al., 2019; Rai et al., 2026). The variation in threshold values among traits further indicates that seedling emergence is relatively more tolerant to salinity, whereas growth and biomass traits are more sensitive, reflecting differences in physiological processes and metabolic demands under stress conditions (Al-Naggar et al., 2023; Bayuelo‐Jiménez et al., 2002).

Cluster analysis further revealed significant genetic variability among quinoa accessions, grouping them into five distinct tolerance categories (HST, ST, MST, SS, and HSS). The highly salt-tolerant group consistently demonstrated superior performance across all evaluated traits, indicating strong adaptive capacity under saline conditions. In contrast, the highly salt-sensitive accession exhibited extreme susceptibility, confirming the broad genetic diversity within quinoa germplasm. Similar clustering-based classifications have been reported in recent studies on various crops, where genotypes are grouped into tolerance classes using integrated indices such as STI and MFV (Wu et al., 2019). Such classification is consistent with previous research demonstrating that combining multiple traits into composite indices improves the accuracy of genotype evaluation under stress conditions. The clear separation of accessions in the dendrogram underscores the robustness of the applied methodology and highlights its potential for identifying elite salt-tolerant genotypes for breeding and cultivation in arid and saline environments (Chaganti and Ganjegunte, 2022; Javed et al., 2022; Tolibova et al., 2025).

## 5 Conclusion

This study demonstrated that salinity stress significantly impairs quinoa seedling emergence and early seedling growth, leading to reduced emergence, delayed emergence, and substantial declines in biomass with increasing salinity. Growth-related traits, particularly fresh and dry biomass, were more sensitive to salinity than seedling emergence parameters, indicating that early seedling development is the most vulnerable stage under salt stress. Significant genotypic variation was observed among the 22 quinoa accessions, enabling their classification into five distinct salinity tolerance groups. Accessions such as PI 634923, PI 698783, PI 698773, PI 698780, and PI 698775 consistently exhibited superior performance under saline conditions, maintaining higher emergence and biomass. In contrast, sensitive accessions showed severe growth inhibition at elevated salinity levels. Salinity increased Na^+^ and Ca²^+^ accumulation while decreasing K^+^ concentration and ionic ratios (K^+^/Na^+^ and Ca^2+^/Na^+^), with tolerant accessions maintaining better ionic balance, suggesting more efficient ion regulation mechanisms. The integration of the salinity tolerance index (STI) and membership function value (MFV) proved an effective approach for evaluating and ranking genotypes, with biomass-related traits serving as reliable indicators of tolerance. The estimated threshold salinity (ECiw ≈ 10.5 dS m^-1^ at 50% STI) serves as a benchmark for the early-stage response of quinoa under greenhouse conditions; however, further validation under field environments and across later growth stages, including seed yield, is required before extrapolating it to practical cultivation. Among the evaluated accessions, PI 634923 and other top-performing accessions identified in this study represent promising candidates for future field validation and breeding programs to improve quinoa’s salinity tolerance. Importantly, the evaluation of previously uncharacterized quinoa accessions revealed additional genetic variability for salinity tolerance under early-stage screening conditions. The applied evaluation approach, integrating the salinity tolerance index (STI) and the membership function value (MFV), proved effective in identifying accessions with superior performance under saline conditions, thereby enabling the detection of promising genotypes for future breeding programs.

Overall, these findings highlight quinoa’s potential as a climate-resilient crop and support its use in salt-affected and water-limited agroecosystems.

## Data Availability

The original contributions presented in the study are included in the article/**Supplementary Material**. Further inquiries can be directed to the corresponding author.
